# Novel lncRNA XLOC_032768 protects against renal tubular epithelial cells apoptosis in renal ischemia–reperfusion injury by regulating FNDC3B/TGF-β1

**DOI:** 10.1080/0886022X.2020.1818579

**Published:** 2020-09-25

**Authors:** Xiangjun Zhou, Yongwei Li, Cheng Wu, Weimin Yu, Fan Cheng

**Affiliations:** a Department of Urology, Renmin Hospital of Wuhan University, Wuhan, China; b Department of Urology, The Affiliated Yantai Yuhuangding Hospital of Qingdao University, Yantai, China

**Keywords:** Acute renal injury, ischemia-reperfusion, hypoxia, cell death, lncRNA

## Abstract

Renal ischemia–reperfusion injury is a leading cause of acute kidney injury, but its underlying mechanism remains poorly understood and effective therapies are still lacking. Here, we identified lncRNA XLOC_032768 as a novel target in renal ischemia–reperfusion injury by analyzing differentially expressed genes of the transcriptome data. PCR results show that XLOC_032768 was markedly downregulated in the kidney during renal ischemia–reperfusion in mice and in cultured kidney cells during hypoxia. Upon induction *in vitro*, XLOC_032768 overexpression repressed the expression of fibronectin type III domain containing 3B (FNDC3B) and tubular epithelial cells apoptosis. Administration of XLOC_032768 preserved FNDC3B expression and attenuated renal tubular epithelial cells apoptosis, resulting in protection against kidney injury in mice. Knockdown of FNDC3B markedly reduced the expression of TGF-β1 and apoptosis of renal tubular cells. Thus, XLOC_032768/FNDC3B/TGF-β1signaling pathway in ischemia–reperfusion injury may be targeted for therapy.

## Introduction

Acute kidney injury (AKI) is very common clinically and has an incidence of 2‰ [1]. This disease poses a major socioeconomic health problem because of its high morbidity and mortality [2]. The prognosis of patients directly depends on the severity of AKI [3]. Ischemia–reperfusion injury (IRI) is one of the common types of AKI [4]. Studies have shown that kidney damage first appears in the proximal renal tubular epithelial cells and are manifested in various forms of cell death; the recovery of renal function depends on the surviving renal tubular epithelial cells through dedifferentiation and proliferation to reconstruct the nephron after AKI [5,6]. Therefore, enhancing the resistance of renal tubular epithelial cells to injury and improving their survival rate are not only beneficial to the repair process after injury but are also of great significance to AKI treatment and prognosis. The definition and classification of apoptosis of proximal renal tubular epithelial cells have been elucidated [7,8]. However, the causes and related mechanisms of apoptosis induced by injury remain unclear. Exploration of the gene-level regulation mechanism is of great clinical value for treatment and even prevention of AKI.

Long noncoding RNAs (lncRNAs) are typically longer than 200 nt [9]. These molecules exert comprehensive effects on biological processes, such as transcription, translation, splicing, and intracellular and extracellular trafficking [10–12]. Studies have shown that lncRNA regulates tissue homeostasis and plays a role in various pathological processes, such as acute and chronic renal diseases [13–15]. Accumulating evidence has indicated the significant roles of lncRNAs in the pathophysiology of AKI [16,17]. We also found that a novel lncRNA XLOC_032768 is repressed after IRI. Whether lncRNA XLOC_032768 is beneficial to the anti-apoptosis ability of renal tubular epithelial cells and the regeneration and repair of kidney and its underlying mechanism remain unclear.

## Materials and methods

### Materials and reagents

C57 mice (weight 20–25 g, male) were obtained from the Changzhou CAVENS Laboratory Animal (Jiangsu, China). HK-2 cells were acquired from the BeNa Culture Collection (Beijing, China). The following materials were also used: anti-FNDC3B antibody (Atlas Antibodies AB, Sweden); anti-TGF-β1 antibody (Boster, Wuhan, China); anti-GAPDH antibody (Service Bio, Wuhan, China); anti-caspase3 antibody (Boster, Wuhan, China); TRIzol (Invitrogen, USA); fetal bovine serum (Gibco, USA); TUNEL Kit (Roche, Shanghai, China); Hoechst (Service Bio, Wuhan, China); Lipofectamine 2000 (Invitrogen, Carlsbad, CA, USA); Bio-Rad S1000 with Bestar SYBR Green RT-PCR Master Mix (DBI Bioscience, Shanghai, China); and AAV GPAAV-CMV-MCS-EF1-ZsGreen1-WPRE (Genomeditech Co., LTD, Shanghai, China).

### Renal IRI model

After 12 h of preoperative fasting, the animals were anesthetized with 10% chloral hydrate solution (9 mL/kg, intraperitoneal injection). The renal pedicle was delineated. The bilateral renal pedicle was blocked by the noninvasive microartery clamp. After 30 min of blocking the renal pedicle, the artery clamp was removed, and blood perfusion was restored. After the operation, the abdominal cavity was closed by layered suture. In the sham operation group, the blood flow along the renal pedicle was not blocked after the renal pedicle was found, and the abdominal cavity was closed after 30 min, 24–48 h of reperfusion after ischemia. Normal saline was used to keep the mice hydrated. After the operation, the mice were kept warm at 24 °C–29 °C and supplemented with water and feed. All processes involving animal treatment were in accordance with the procedures of the Ethical Committee for Animal Experimentation, Renmin Hospital of Wuhan University. All procedures were performed according to the guidelines for the care and use of laboratory animals.

### Library construction and sequencing

Total RNA was extracted from all renal tissues by using TRIzol Reagent (Ambion) following the manufacturer’s instructions. Several processes were performed to detect the total RNA of the sample. Agarose gel electrophoresis was used to analyze the degree of RNA degradation and determine possible contamination. The purity of the RNA was detected by Nanodrop (od260/280 ratio). Qubit was used to accurately quantify RNA concentration. The RNA integrity was accurately detected by Agilent 21. After the samples were tested to be qualified, a small RNA sample pre-kit was used to construct the library. The total RNA was used as the starting sample by using the special structures of the 3′- and 5′-ends of the small RNA (the 5′-end had a complete phosphate group, whereas the 3′-end had a hydroxyl group). The small RNA was directly spliced at both ends and then reverse transcribed into a complementary DNA (cDNA). After PCR amplification, the target DNA fragment was separated by PAGE, and the recovered cDNA library was obtained by gel cutting. After the library construction was completed, Qubit2.0 was used for preliminary quantification, the library was diluted to 1 ng/UL, and Agilent 2100 was used to detect the insert size of the library. After the insert size met the expectation, Q-PCR analysis was performed. The effective concentration (more than 2 nm) of the library was quantified accurately to ensure the quality of the library. Different libraries were pooled according to the requirements of effective concentration and target offline data volume. Hiseq/Miseq sequencing was performed (ABlife Inc., Wuhan, China).

### Cell culture and plasmid transfection

HK-2 cells were cultured in a culture flask with Dulbecco’s modified Eagle’s medium supplemented with 10% fetal bovine serum at 37 °C in a humidified atmosphere of 5% CO_2_ and 95% air or an anoxic environment (94% N_2_, 5% CO_2_ and 1% O_2_). Cells (1 × 10^4^) were seeded in 24-well culture plates. After the cells reached 70% confluence, the vector was transfected into the HK-2 cells by using Lipofectamine 2000 according to the manufacturer’s protocol. The cells were then incubated at 37 °C for 24 h. Glyceraldehyde-3-phosphate dehydrogenase (GAPDH) was used as the control gene to assess the effects of the target lncRNA overexpression. cDNA was synthesized by standard procedures, and RT-qPCR analysis was performed on Bio-Rad S1000 with Bestar SYBR Green RT-PCR Master Mix. The concentration of each transcript was then normalized to the GAPDH mRNA level by using 2^−ΔΔCT^ method.

### Generation of target FNDC3B silenced cells

We selected and mixed three effective short-hairpin RNAs (shRNAs) to knock down the expression of FNDC3B (the target sequences were as follows: FNDC3B shRNA1: 5′-GCAGGTTATTCTCGTTCAA-3′; FNDC3B shRNA2: 5′-GCTTACTACCCACCTGTTA-3′; and FNDC3B shRNA3: 5′-GCAGCTGCACAACAGTATA-3′) by infection with 293 T-produced lentivirus. For the lentivirus production, the supernatant of 293 T culture was harvested after transfection with shRNA vectors. The targeted cells were then incubated with lentiviruses for 24 h with 2 μg/mL polybrene (Sigma-Aldrich, MO, USA).

### Adeno-associated virus vector (AAV) design, production, and delivery

AAV GPAAV-CMV-MCS-EF1-ZsGreen1-WPRE (Genomeditech Co., LTD, Shanghai, China) and XLOC_032768 plasmids were cotransfected into AAV-293 cells by using the HG transgene reagent. After 10–12 h of transfection, an enhancing buffer was added, and the fresh culture medium was changed after 8 h. After 48 h of continuous culture, the virus particles were collected. For systemic administration, GPAAV-CMV-lncRNA XLOC_032768-EF1-ZsGreen1-WPRE were injected *via* the tail vein to mice at 2 × 10^11^ vg 2 days before renal ischemia.

### Histopathological examination of kidney

The kidney was fixed in 10% formalin solution, embedded in paraffin, and made into 4 mm sections. The samples were stained with conventional hematoxylin–eosin (HE) and evaluated according to the Rabb semiquantitative pathological evaluation scoring (maximum, 4 points) [normal renal morphology, 0 point; minimal necrosis (< 5% tubular necrosis), 1 point; mild necrosis (5%–25% tubular necrosis), 2 points; moderate necrosis (25%–75% tubular necrosis), 3 points; and severe necrosis (> 75%), 4 points]. Renal tubular apoptosis was examined by TUNEL assay by using a detection kit according to the manufacturer’s instruction. TUNEL-positive nuclei were identified by microscopy.

### Hoechst staining

Hoechst-stained apoptotic cells were brighter than normal cells. Hoechst was added to the cells, and the cells were then incubated at 37 °C for 20 min. The percentage of the Hoechst-positive cell was calculated using the Image-Pro Plus 6 analysis software.

### Western blot analysis

Whole cell or tissue lysate was collected in 2% SDS buffer. Protein concentration was measured in the supernatant by using BCA reagent. Concentrated and separating gel by using 30 g of protein samples. Gel electrophoresis was performed in PVDF membrane in 2% bovine serum albumin (BSA) for 1 h at room temperature. First antibody (FNDC3B, caspase3 and TGF-β1 antibody) incubation was performed at 4 °C overnight. Second antibody incubation was performed at room temperature for 1 h. The cells were exposed to the exposure solution and imaged in a gel imaging system. The gray value of the electrophoresis strip was analyzed according to Quantity One software.

### Real-time RT-PCR

Total RNAs were isolated from the HK-2 cells or kidney tissues. Nanodrop 2000 was used to detect the RNA concentration and purity. Reverse transcription was performed using the following reagents: 4 μL of 5× reaction buffer, 2 μL of 10 mm dNTP mix; 1 μL of RiboLock RNase inhibitor (20 u/μL), and 1 μL of Revertai M-MuLV reverse transcriptase (200 U/μL). The solution was well mixed with a gun suction. For the Q-PCR, 0.2 mL PCR tube was used. The following reaction system was prepared: 2× qPCR Mix, 12.5 μL; 7.5 μM gene primer, 2.0 μL; RT product, 2.5 μL; and ddH_2_O, 8.0 μL. PCR amplification was conducted as follows: predenaturation at 95 °C for 10 min (40 cycles), 95 °C, 15 *s* → 60 °C, 60 s. The melting curve was determined at 60 °*C* → 95 °C. The samples were heated at 0.3 °C every 15 s. The quantified values were shown as 2^−ΔΔCt^ values. The sequences of the primer were as follows: for the mouse XLOC_032768, forward primer was 5′- GACTAGATGCTGCTGCTGGA-3′, and reverse primer, 5′- AGGCTTCTTGGTGTCAGTAGG −3′; for the human XLOC_032768, forward primer was 5′- CATTGCCGACAGCACAACATAC −3′, reverse primer was 5′- GCCTATTTAGCAGCCCACCTC −3′; for the mouse GAPDH, forward primer was 5′- CCTCGTCCCGTAGACAAAATG −3′, and reverse primer was 5′-TGAGGTCAATGAAGGGGTCGT-3′; and for the human GAPDH, forward primer was 5′-CTACCCACGGCAAGTTCAAC-3′, and the reverse primer was 5′-CCAGTAGACTCCACGACATAC-3′.

### Statistical analysis

Significant *p* values of differential expression were calculated by Student’s *t*-test when only two groups were compared. Hierarchical clustering method was used to cluster the differently expressed pattern genes. χ^2^-test was used to analyze the differential expression. All values are presented as mean ± SD. *p* Values < 0.05 were considered statistically significant. All statistical analyses were performed by the R software.

## Results

### XLOC_032768 was downregulated in the kidney during renal IRI in mice and in the cultured kidney cells

**Figure 1.**
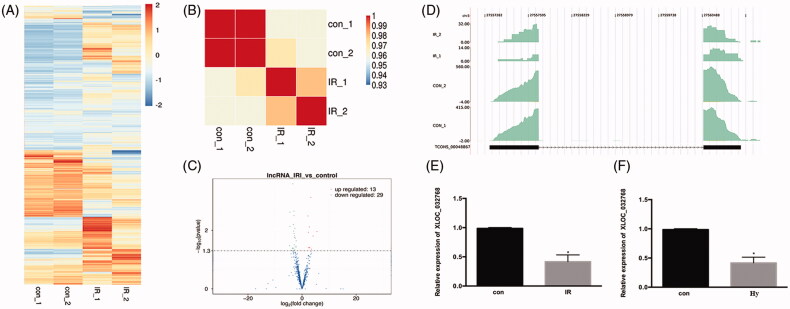
LncRNA XLOC_032768 was downregulated in the kidney during renal ischemia-reperfusion in mice and in the cultured kidney cells. (A) heat map showed the differentially expressed genes after ischemia–reperfusion injury (IRI). (B) Sample correlation analysis revealed that the IRI group could be clearly distinguished from the control group. (C) Volcano plot presented the differentially expressed lncRNAs between the IRI and control groups. (D) Read density plot showed the repressed expression level of XLOC_032768 in cisplatin-treated samples. (E) and (F) Real-time PCR analysis of XLOC_032768 in the IRI kidney (*n* = 3 for each control group; *n* = 6 for each cisplatin treatment group) and HK-2 cells treated with hypoxia (*n* = 3). The data in (E, F) are expressed as means ± SD. *p < 0.05 vs. sham or control group.

We tested the global expression profile alteration for the mRNA genes by the transcriptome profile from the mouse model (Figure 1(A)). Sample correlation analysis and Volcano plot revealed the differentially expressed genes between the IRI samples and controls (Figure 1(B,C)). The IRI group could be clearly distinguished from the control group. The density plot of the reads showed that a novel and highly expressed multiple-exonic XLOC_032768 was repressed in ischemia injury (Figure 1(D)). Real-time PCR analysis revealed that the expression level of XLOC_032768 was markedly inhibited in the renal tissue (Figure 1(E)) and in HK-2 cells exposed to hypoxia (Figure 1(F)).

### Overexpression of XLOC_032768 decreased the apoptosis of HK-2 cells by hypoxia treatment

**Figure 2.**
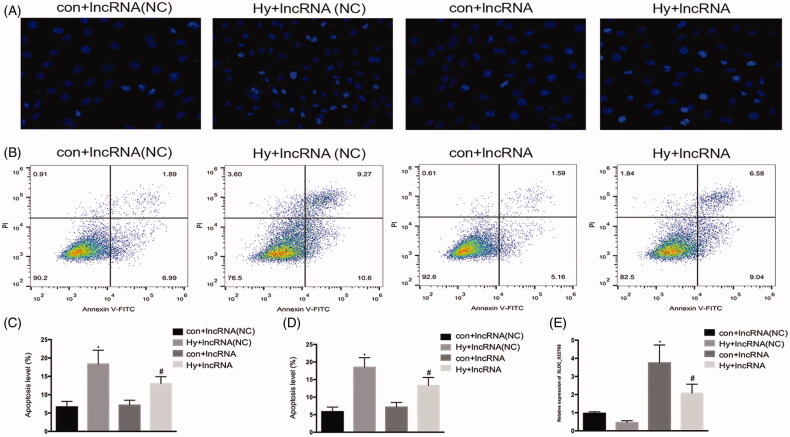
Overexpression of XLOC_032768 showed decreased apoptosis of HK-2 cells by hypoxia treatment. HK-2 cells were transfected with XLOC_032768 by using Lipofectamine 2000 and then treated with hypoxia for 48 h. (A) Hoechst analysis determined HK-2 cells apoptosis. (B) Annexin V flow cytometry analysis determined HK-2 cell apoptosis. (C) Quantitative analysis of the percentage of apoptotic cells by flow cytometry between the groups. (D) Bar chart from Hoechst staining shows the ratios of apoptosis cell numbers between the groups. (E) Bar plot showing the RT-qPCR results of lncRNA XLOC_032768 expression and negative control in normal and hypoxia samples, respectively. The data is expressed as means ± SD (*n* = 3). *p < 0.05 vs. control + lncRNA (NC) group; ^#^*p* < 0.05 vs. hypoxia + lncRNA (NC) group.

We examined the role of lncRNA XLOC_032768 in the hypoxia-induced injury of renal proximal tubular epithelial cells by using the *in vitro* model of HK-2 cells. XLOC_032768 was successfully transfected into HK-2 cells as indicated by the results of RT-qPCR (Figure 2(E)). Hoechst staining observation showed that the overexpression of XLOC_032768 substantially reduced the apoptosis of HK-2 cells after hypoxia (Figure 2(A,C)). The observation was verified by Annexin V flow cytometry analysis (Figure 2(B,D)). These results suggested that XLOC_032768 may rescue tubular cell injury and death during hypoxia treatment.

### Administration of XLOC_032768 attenuated renal dysfunction and renal tubular cell injury

**Figure 3.**
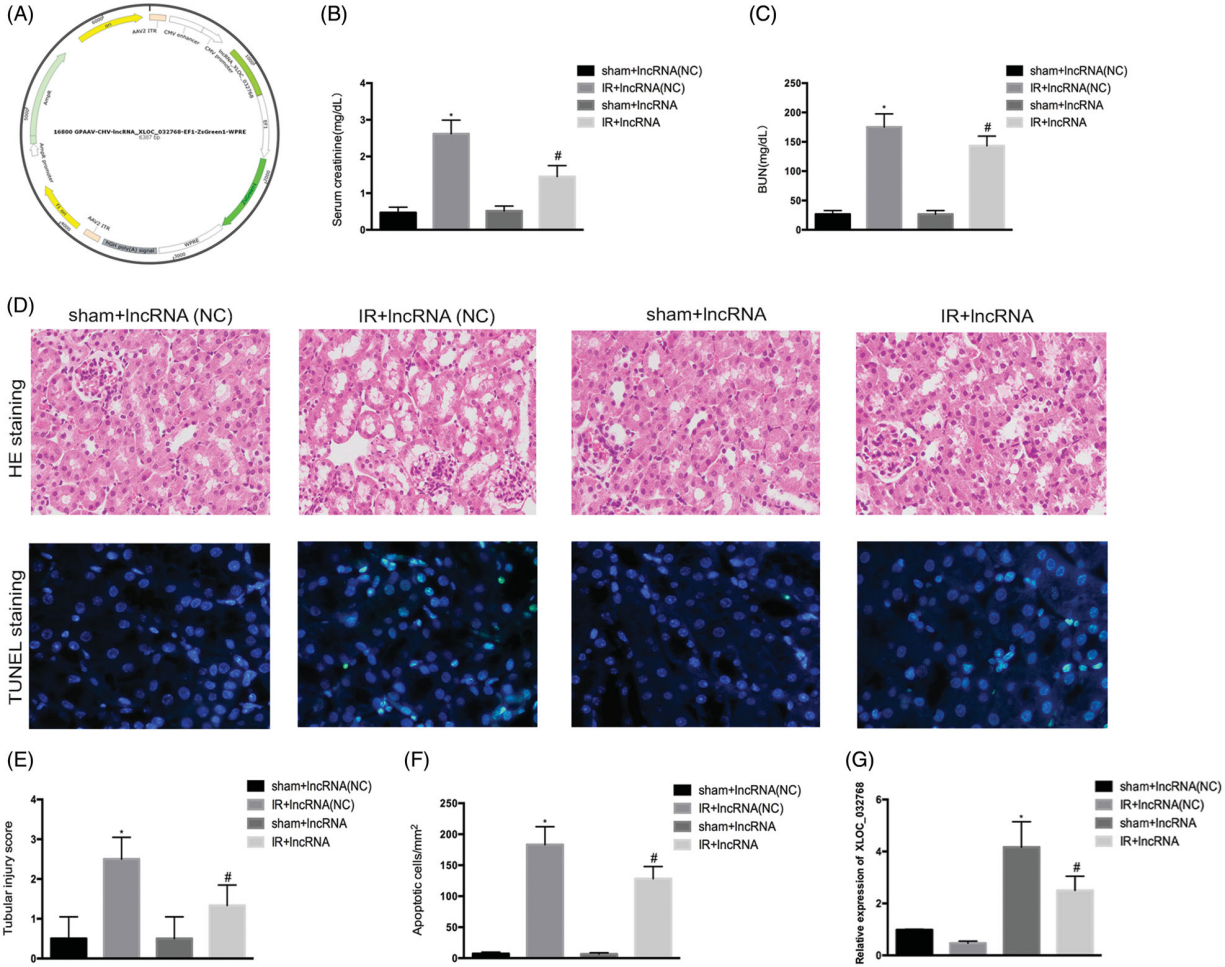
Administration of XLOC_032768 attenuated renal dysfunction, morphological damage, and renal tubular cell apoptosis. (A) Adeno-associated virus (AAV) with XLOC_032768 plasmids were injected *via* the tail vein followed by ischemia-reperfusion. (B) and (C) Serum samples were evaluated for blood urea nitrogen (BUN) and serum creatinine to indicate the decrease of renal function in cisplatin-treated mice. (D) Renal tissues were collected for HE staining to record tubular injury. (E) TUNEL analysis of renal tubular apoptosis. (F) Semi-quantify tubular damage. (G) Semi-quantify apoptosis. (H) Bar plot showing the RT-qPCR results of XLOC_032768 expression in normal and ischemia-reperfusion samples, respectively. The data is expressed as means ± SD (*n* = 3). *p < 0.05 vs. sham + lncRNA (NC) group; ^#^*p* < 0.05 vs. IR + lncRNA (NC) group.

We examined the possible involvement of lncRNA XLOC_032768 in renal IRI. GPAAV-CMV-lncRNA XLOC_032768-EF1-ZsGreen1-WPRE was injected *via* the tail vein into the kidney (Figure 3(A)). PCR analysis confirmed the significant increase of XLOC_032768 in the renal tissue (Figure 3(H)). Administration of lncRNA XLOC_032768 decreased the levels of BUN and serum creatinine in mice (Figure 3(B,C)). Renal histology revealed significantly less tissue damage after lncRNA XLOC_032768 treatment (Figure 3(D,F)). Moreover, TUNEL analysis showed reduced apoptosis with lncRNA XLOC_032768 treatment (Figure 3(E,G)). These results suggested that XLOC_032768 treatment may attenuate renal dysfunction and rescue tubular cell injury during IR.

### FNDC3B could be the target gene of XLOC_032768 in mice and in the cultured kidney cells

**Figure 4.**
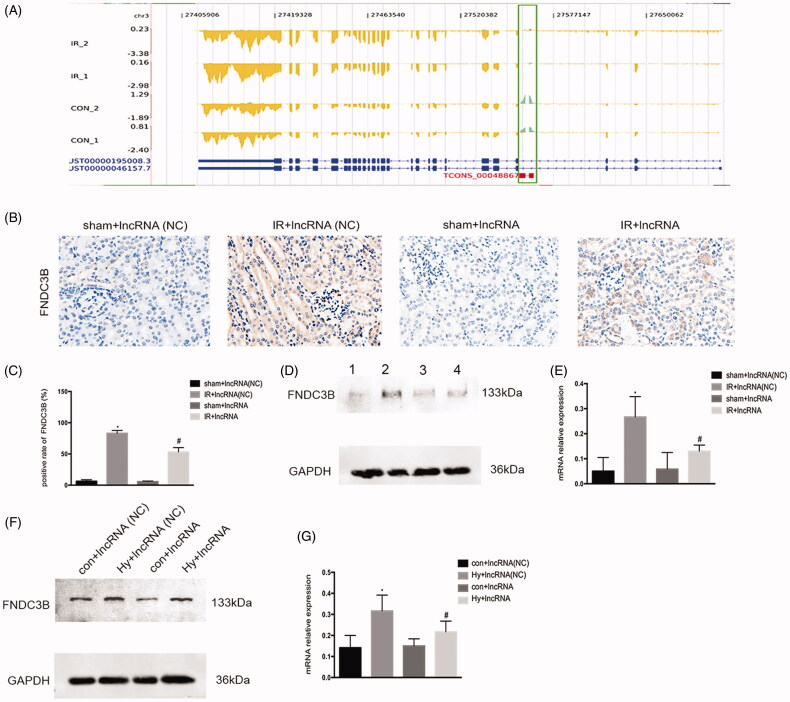
FNDC3B could be the target gene of XLOC_032768 in mice and in the cultured kidney cells. (A) The expression and position relationship of XLOC_032768 and FNDC3B. The blue gene structure is FNDC3B, the green box is the location of XLOC_032768, the yellow structure indicates the expression abundance of FNDC3B, and the green sequencing fragment accumulation results indicate the expression abundance of XLOC_032768. (B) Representative images of renal histology by Immunohistochemistry. (C) Semiquantified FNDC3B. (D) Western blot showing the repressed expression level of FNDC3B in the kidney after ischemia-reperfusion under XLOC_032768 overexpression. (E) Semiquantified FNDC3B. (F) Western blot showing the repressed expression level of FNDC3B in renal tubular cells after hypoxia treatment under XLOC_032768 overexpression conditions. (G) Semi-quantified FNDC3B. Data were expressed as mean ± SD (*n* = 3). *p < 0.05 vs. sham + lncRNA (NC) group or control + lncRNA (NC) group; ^#^*p* < 0.05 vs. IR + lncRNA (NC) group or hypoxia + lncRNA (NC) group.

LncRNAs play an important role in the regulation of gene expression. As shown in Figure 4(A), XLOC_032768 have a significant negative correlation with FNDC3B from the expression and position. Immunohistochemistry indicated the FNDC3B located in renal tubular, the expression of FNDC3B was repressed in the kidney after ischemia–reperfusion under XLOC_032768 overexpression (Figure 4(B,C)). Western blots further confirmed the repressed expression level of FNDC3B in the kidney after ischemia–reperfusion (Figure 4(D,E)) and in the renal tubular cells after hypoxia treatment (Figure 4(F,G)) under XLOC_032768 overexpression. These results suggested that FNDC3B could be the target gene of XLOC_032768.

### Knockdown of FNDC3B markedly reduced the apoptosis of renal tubular cells

**Figure 5.**
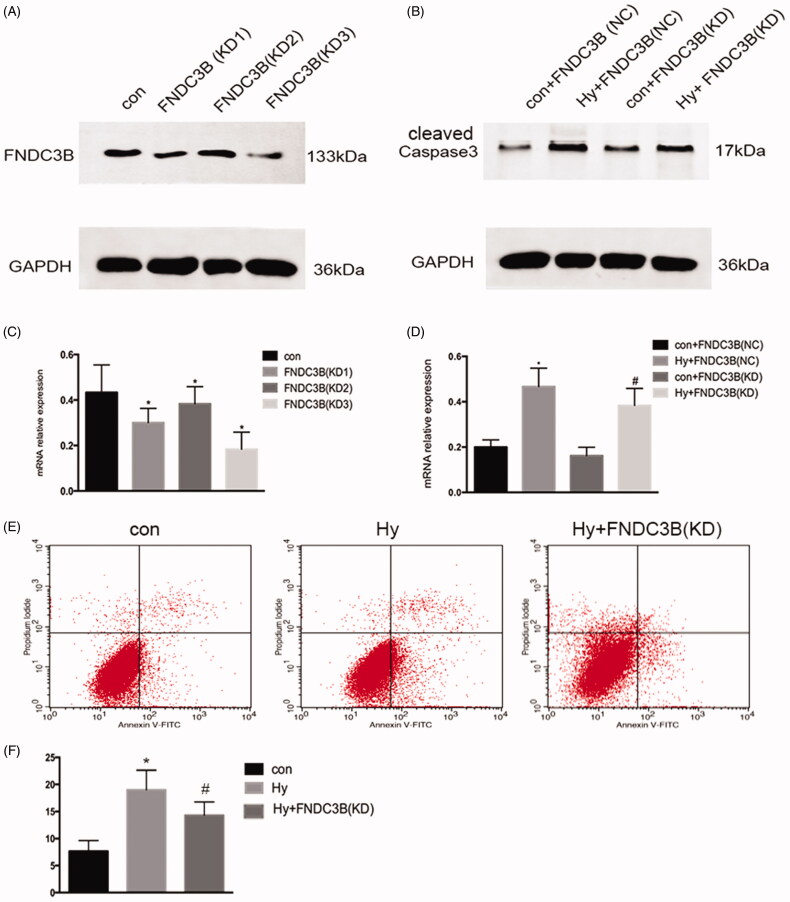
Knockdown of FNDC3B markedly reduced the apoptosis of renal tubular cells. We generated HK-2 cells with stably knocked down FNDC3B expression using effective short-hairpin RNAs. (A) Western blot was performed to assess the FNDC3B knockdown efficiency. (B) Western blotting was performed to assess the expression level of Caspase3 with stably knocked down FNDC3B in HK-2 cells. (C) Semi-quantify FNDC3B. (D) Semi-quantify Caspase3. (E) Flow cytometry analysis of the effect of inhibition of FNDC3B on HK-2 cell apoptosis. (F) Quantitative analysis of the percentage of apoptotic cells by flow cytometry. Data were expressed as mean ± SD (*n* = 3). *p < 0.05 vs. control or control + FNDC3B (NC) group; ^#^*p* < 0.05 vs. hypoxia + FNDC3B (NC) group.

We generated HK-2 cells with stably knocked down FNDC3B expression using effective short-hairpin RNAs (Figure 5(A,C)). The knockdown of FNDC3B markedly reduced the apoptosis of renal tubular cells (Figure 5(B,D)). Moreover, subsequent flow cytometry analyses revealed that the number of apoptotic cells in the knockdown of FNDC3B group was lower than that in the hypoxia group alone (Figure 5(E,F)). These results suggested that FNDC3B was associated with the apoptosis of renal tubular cells.

### XLOC_032768/FNDC3B downregulated the expression of TGF-β1 in cultured epithelial cells

**Figure 6.**
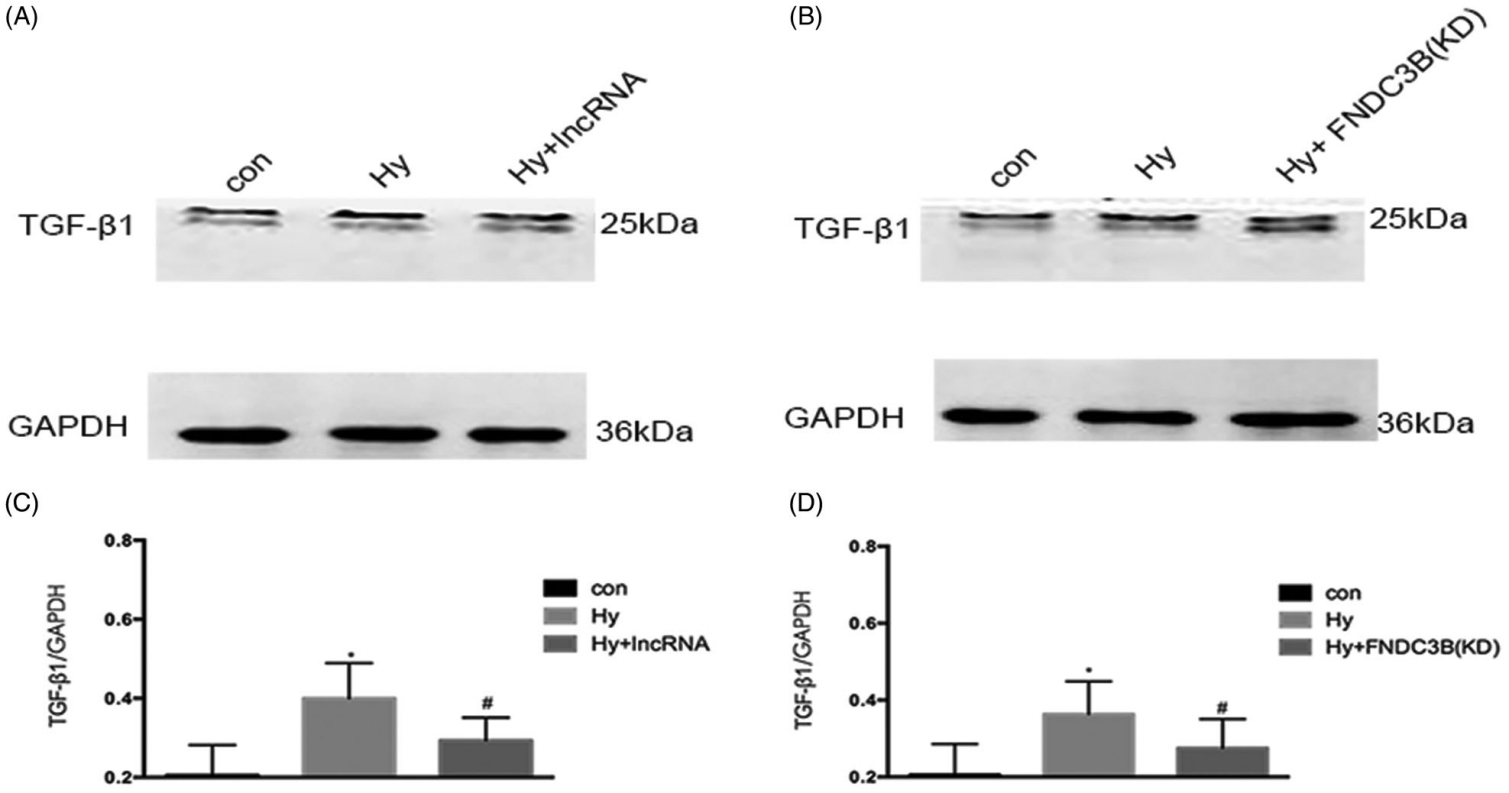
XLOC_032768/FNDC3B downregulated the expression of TGF-β1 in cultured epithelial cells. (A) Western blotting was performed to assess the expression level of TGF-β1 with overexpression XLOC_032768 in HK-2 cells. (B) Western blotting was performed to assess the expression level of TGF-β1 with stably knocked down FNDC3B in HK-2 cells. (C) Semi-quantify TGF-β1. (D) Semi-quantify TGF-β1. Data were expressed as mean ± SD (*n* = 3). *p < 0.05 vs. control; ^#^*p* < 0.05 vs. hypoxia group.

As shown in Figure 6, western blot analysis demonstrated that hypoxia resulted in a marked increase in the level of TGF-β1. Compared with the hypoxia group, the hypoxia plus XLOC_032768 group demonstrated significant reductions in TGF-β1 expression of HK-2cells (Figure 6(A,C)), knockdown of FNDC3B also led to a significant decrease in TGF-β1 levels in hypoxia epithelial cells (Figure 6(B,D)). These results suggested that TGF-β1could be downstream gene of XLOC_032768/FNDC3B.

## Discussion

In this study, we demonstrated through *in vivo* mouse and *in vitro* HK-2 cell models that a novel lncRNA XLOC_032768 protects against ischemic AKI. Mechanistically, lncRNA XLOC_032768 may attenuate hypoxia-induced renal tubular cell apoptosis *via* FNDC3B/TGF-β1.

Accumulating evidence has indicated the significant roles of lncRNAs in the pathophysiology of AKI, and the crosstalk between lncRNA and AKI has been widely reported in recent years [18–20]. LncRNAs are involved in the progression of AKI by regulating many important factors. These molecules may interact with all components of the cellular machinery, such as protein, DNA, and RNA [21,22]. Metastasis-associated lung adenocarcinoma transcript 1 (MALAT1), which has been initially identified as the most highly induced lncRNA gene in the kidney of hypoxic mice, was proposed to be activated by hypoxia inducible factor (HIF)-2 and postulated to function in renal proximal tubular [23,24]. MALAT1 expression has been suggested to inhibit the hypoxia-induced inflammatory response through the NF-κB pathway. LncRNA colorectal neoplasia differentially expressed (CRNDE) protected against sepsis-induced kidney injury by inhibiting the proliferation and promoting the apoptosis of renal cells *via* the miR-181a-5p/PPARα pathway [25]. LncRNA NEAT1 promotes hypoxia‐induced renal tubular epithelial apoptosis by downregulating miR‐27a‐3p [26]. In the current study, we provide the first evidence of the regulation and functional role of XLOC_032768 in ischemic AKI. This lncRNA was downregulated *in vivo* in ischemia-reperfused kidneys and *in vitro* during the hypoxic incubation of cultured renal proximal tubular cells. Upon induction, lncRNA XLOC_032768 may play a cytoprotective role for renal cell apoptosis by suppressing FNDC3B. XLOC_032768, which is a highly expressed multiple-exonic lncRNA and first reported to decrease in response to ischemia and hypoxia in the kidney. To verify the induction of XLOC_032768 and identify its functional form in ischemic AKI, we conducted QRT-PCR by using specific Taqman probes to XLOC_032768. The analysis revealed the induction of XLOC_032768 during renal IRI. Further functional study showed that the overexpression of XLOC_032768 decreased apoptosis during hypoxic incubation.

Under various pathological conditions, lncRNA XLOC_032768 may target different downstream genes. In this study, we verified FNDC3B as a target of lncRNA XLOC_032768. FNDC3B is also known as an important oncogenic driver gene [27]. Consistent with its oncogenic role in multiple cancer types, the overexpression of FNDC3B could malignantly transform mammary and kidney epithelial cells and hepatocytes [28]. FNDC3B may trigger either apoptotic or survival signals by the recruitment of different sets of molecules [29]. Overexpression of FNDC3B in HCC cell lines enhanced cell migration and invasion, knockdown of FNDC3B using shRNA reduced tumor nodule formation in intra- and extra-hepatic metastasis [30]. In our study, knockdown of FNDC3B led to the protection of renal tubular cells from apoptosis, suggesting that FNDC3B is a proapoptosis factor in hypoxic renal tubular cells.

TGF-β signaling has been shown to be beneficial or detrimental to the tubular response to AKI. TGF-β may facilitate proximal tubule repair by accelerating de-differentiation of surviving epithelial cells [31]. TGF-β can also increase proximal tubule apoptosis, which might play an important pathologic role in ischemic, septic, and toxin-induced forms of AKI [32,33]. Inhibition of TGF-β signaling in proximal tubular cells has been reported to attenuate kidney injury [34]. In this study, we confirmed that XLOC_032768/FNDC3B downregulated the expression of TGF-β1 in cultured epithelial cells, TGF-β1could be downstream gene of XLOC_032768/FNDC3B in renal epithelia cells. Recent study have reported that upregulated FNDC3B during hypoxia could induce epithelial–mesenchymal transition and activate several pathways, such as PI3-kinase/Akt, Rb1, and TGF-β signaling in tongue squamous carcinoma cells [35].

In conclusion, the expression of lncRNA XLOC_032768 was inhibited during ischemic AKI and hypoxic incubation of kidney cells. Treatment with XLOC_032768 protected kidney cells and tissues against injury. The protective effect of XLOC_032768 may involve the target gene FNDC3B/TGF-β1.
